# Everybody's Page

**Published:** 1888-11-17

**Authors:** 


### Everybody's Page.

Foaming beer has been suggested as a vehicle for administering castor-oil. The beer is said to prevent sickness, as well as to disguise the taste.

The amount of food, liquid and solid, which the average man consumes in his seventy years is calculated at no less than eighty tons.

Telegraph operators, it seems, are developing a disease of their own. One or two cases have recently occurred in which the finger-nails have dropped off, one after another. This affection is supposed to be due to the constant hammering and pushing with the finger-ends required by the working of the Norse system of telegraphy.

The French nation has determined to imitate the English in one important particular, the introduction of athletic sports into their schools. How long it will take to train up Gustave and Achille in the way that goes to make a W. G. Grace no man knoweth, but certainly the school authorities are taking a step in the right direction for a "nation of soldiers".

Mr. Carl Rosa has been speaking to the inhabitants of Liverpool of the duty of developing a love of music in their midst by forming a local orchestra, but the proposal has not met with much favour. One civic dignity made a calculation of the amount that would necessarily be expended in salaries, and "Why!" he exclaimed in horror, "it would be more than we pay for our lunatic asylum."

Among new experiments in therapeutics, will anyone try the Chinese remedy for croup? Here is the prescription: Collect from old walls seven nests of large-sized spiders, two of which, at least, should contain live spiders. Make them into a paste, with thirty grains of alum previously dissolved. Then, after it is well mixed, reduce the paste to ashes, and apply it hot to the throat. This, it is said, will effect an instantaneous cure of the disease, however severe the attack may be.

Medical science, which is effecting a sure, though gradual, conquest over so many diseases, seems to fail, in America, at least, to overtake the ravages of pneumonia. The rate of mortality from that disease seems to be increasing in alarming fashion. Taking the statistics of the Pennsylvania Hospital, it appears that in it the death-rate from pneumonia was 6¼ per cent. in the years 1845, 46, 47, but rose to 18½ per cent. in 1865, 66, 67, and to the very serious proportion of 31 per cent. in 1884, 85, 86. In short, nearly one-third of the patients who die in that institution are killed by pneumonia, and though hospital cases are often more severe than those met with in private practice, there can be no doubt that these statistics imply a serious increase in the spread of pneumonia and the mortality that arises from it.

Dr. C. S. Ray and Dr. J. G. Adami publish in the *National Review* for this month their paper on waist-belts and stays, which so surprised the members of the British Association at Bath. Most women will appreciate their argument that the fashion of wearing a girdle, which has prevailed through so many civilizations, from the Egyptian to our own, and has survived innumerable changes of fashion, must depend on something more than mere caprice and vanity. They say that a slight compression of the abdominal organs adds to the amount of blood available for other parts of the organism, and is therefore useful for periods of increased mental or physical activity. If it be true that even the men of the Elizabethan age wore corsets, we must admit some foundation for the theory. Drake, Raleigh, and their compeers were weaklings in neither body nor mind.

The effect of any extra atmospheric pressure on the ear, beyond the average sixteen pounds to the inch, is both painful and dangerous, and workmen whose duties force them to undergo it—for example, workers on tunnels and bridges, where the pressure in the submarine caissons is of from twenty to forty pounds to the square inch—are apt to suffer not only from deafness, but from purulent disease of the ear. It appears, however, that men of strictly temperate and healthy habits are less liable than others to trouble of this sort.

The *Medical Record* says: "Some individual has suggested that physcians adopt a peculiar style of dress in order to distinguish them from the vulgar herd. We doubt whether a peaked cap with bells would render the originator of this scheme more conspicuous among his fellow men." Is not the *Record* unnecessarily severe? The soldier is proud of his coat, the lawyer "fancies himself" in his wig and gown, the clergyman feels a special responsibility upon him when he dons his gown or surplice, and, to come nearer home, the nurse looks upon her uniform as something almost sacred. It is to her at once a safeguard and an inspiration, "Do you think I would disgrace my uniform?" is her most indignant protest against an unjust accusation. If the wearing of a uniform of some kind were to impress upon medical men—we don't say it assuredly would—an added sense of vocation and responsibility, a great deal might be said in favour of it.

A week or two ago twelve cyclists went from London to Derby. "There's nothing wonderful in that," says the unthinking reader. Ah! but there is; for of the twelve there were ten who were blind—pupils from the Royal Normal College for the Blind. They travelled on two Rudge machines, one constructed to carry four persons and the other capable of seating eight, each of which had a seeing rider in the first seat to act as steerer. The journey, the idea of which was a happy inspiration that came to Dr. Campbell, the president of the college, took three days to perform, and excited great interest all along the road. When the party neared Derby they were joined by a large body of local cyclists, who had come out to act as escort to their blind brethren. Cycling is one of the means most recently tried for providing the blind with exercise, and thus preventing the physical weakness and the resulting morbid habit of mind that so frequently characterises them.

M. De Flaix brings forward some rather surprising statistics on the influence of alcohol on morality and health. He says that in those departments of France where the consumption of alcohol is small the birth-rate is less and the mortality greater than elsewhere; and neither crime nor suicide is in proportion to alcoholic consumption. Comparing different nations, he says: "France consumes less alcohol than the United Kingdom; its birth-rate is less, and its mortality, criminality, and suicide rates are greater. Italy consumes very little alcohol; its criminality is appalling. Spain consumes three times less alcohol than Italy; its criminality is double. Sweden, Denmark, and Norway, with a population of about one third, consume four times the quantity of alcohol consumed in Italy, and yet the criminality of the former is very small, while that of the latter is appalling." M. De Flaix seems to think that the consumption of alcohol is in direct correlation with the amount of crime in the countries he compares. Is it not rather a question of blood? The phlegmatic Norseman can consume more alcohol, and be less excited by it, than the more emotional Latin race. Nevertheless, M. De Flaix is as much justified in his conclusions as are those enthusiasts who too rashly associate alcohol and crime.

